# Supplementary material for: The effects of genital myiasis on the diversity of the vaginal microbiota in female Bactrian camels
Source: BMC Vet Res. 2022 Mar 5;18:87. doi: 10.1186/s12917-022-03189-5 (PMC8897907; doi:10.1186/s12917-022-03189-5)
Supplement: Supplementary file 5 — Additional file 5. [file 12917_2022_3189_MOESM5_ESM.zip › MPL201709200_16s_yy/Treat1/B13_bdiv/NMDS_unweighted.pdf]

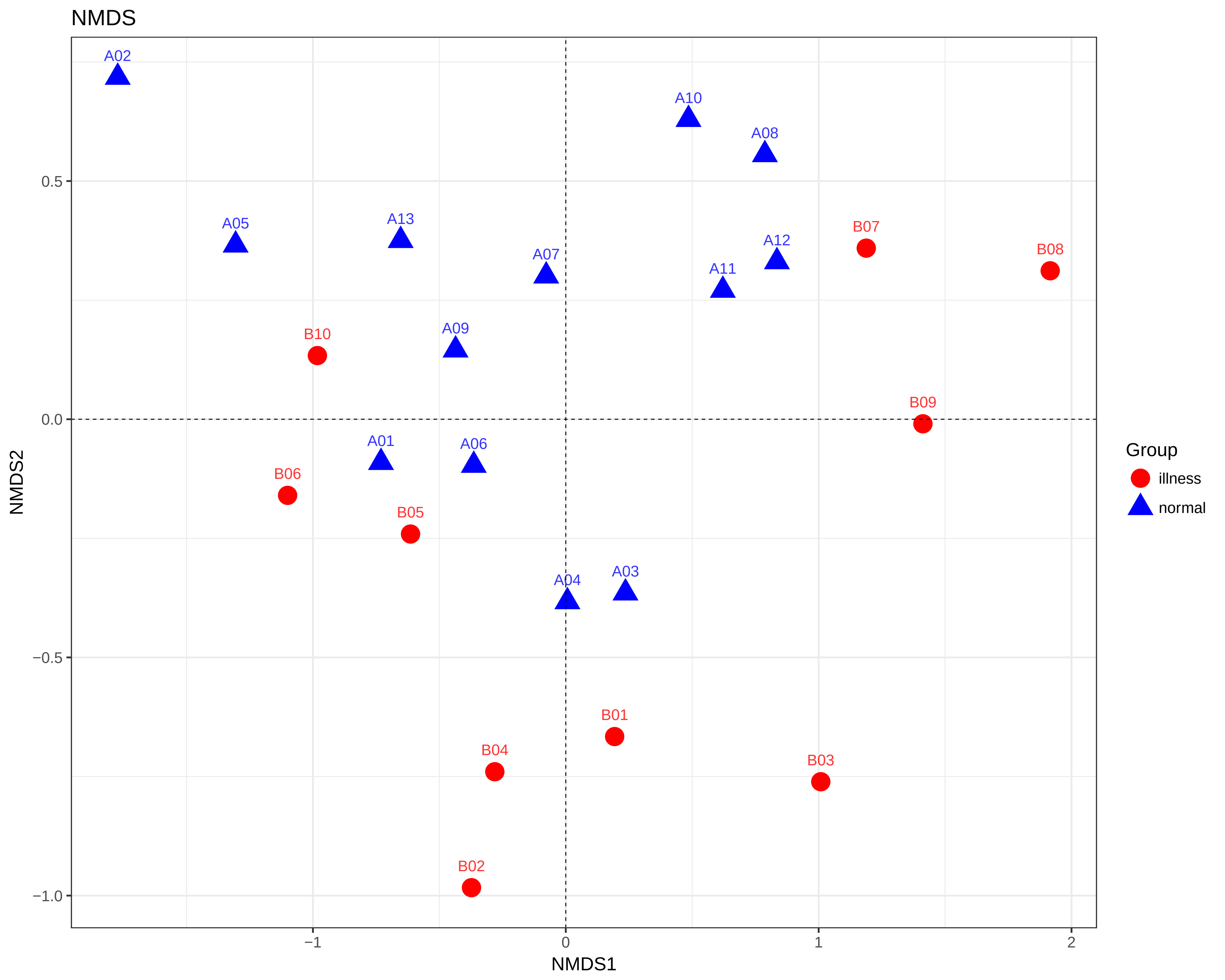

NMDS
A02
A10
A08
A05
A13
A07
A12
A11
B07
B08
B10
A09
B09
A01
A06
B06
B05
A04
A03
B01
B04
B03
B02
0.5
0.0
−0.5
−1.0
−1
0
1
2
NMDS1
NMDS2
Group
illness
normal
